# Supplementary material for: Changing Prevalence of AIDS and Non-AIDS-Defining Cancers in an Incident Cohort of People Living with HIV over 28 Years
Source: Cancers (Basel). 2023 Dec 22;16(1):70. doi: 10.3390/cancers16010070 (PMC10777974; doi:10.3390/cancers16010070)
Supplement: Supplementary file 1 [file cancers-16-00070-s001.zip › cancers-2721723-supplementary.docx]

**Supplementary materials**

**Table S1.** Characteristics of the study population, cancer treatment and outcome of the entire cohort and by decade of cancer diagnosis (all proportions are intended as column percentage).

| **Characteristics** | | **Overall N= 289** | **First decade (1996 - 2003) N = 75** | **Second decade (2004 - 2013) N = 90** | **Third decade (2014 - 2023) N = 124** | **p-value** |
| --- | --- | --- | --- | --- | --- | --- |
| **Age (at cancer diagnosis)** | mean (SD) | 49.5 (12.2) | 41.6 (10.4) | 49.4 (10.7) | 54.4 (11.7) | **<0.0001** |
|  | Med [IQR] | 49.0 [41.0;58.0] | 39.0 [34.0;45.0] | 48.5 [42.2;55.0] | 55.0 [46.0;61.0] |  |
| **Sex, n (%)** | Female | 64 (22.1%) | 10 (13.3%) | 22 (24.4%) | 32 (25.8%) | 0.0994 |
|  | Male | 225 (77.9%) | 65 (86.7%) | 68 (75.6%) | 92 (74.2%) |  |
| **Nationality, n (%)** | Italian | 263 (91.0%) | 69 (92.0%) | 84 (93.3%) | 110 (88.7%) | 0.4760 |
|  | Foreign | 26 (9.0%) | 6 (8.0%) | 6 (6.7%) | 14 (11.3%) |  |
| **Tumor type, n (%)** | ADC | 120 (42.0%) | 58 (77.3%) | 35 (38.9%) | 27 (22.3%) | **<0,0001** |
|  | NADC | 166 (58.0%) | 17 (22.7%) | 55 (61.1%) | 94 (77.7%) |  |
| **Tumor, n (%)** | Kaposi | 63 (21.8%) | 30 (40.0%) | 18 (20.0%) | 15 (12.1%) | **<0.0001** |
|  | NHL | 58 (20.1%) | 28 (37.3%) | 18 (20.0%) | 12 (9.7%) |  |
|  | HCC | 21 (7.3%) | 1 (1.3%) | 7 (7.8%) | 13 (10.5%) |  |
|  | Anal cancer | 19 (6.6%) | 2 (2.7%) | 5 (5.6%) | 12 (9.7%) |  |
|  | Non-melanoma skin cancer | 16 (5.5%) | 1 (1.3%) | 3 (3.3%) | 12 (9.7%) |  |
|  | Neoplasms of the oral cavity, pharynx, and salivary glands | 14 (4.8%) | 2 (2.7%) | 7 (7.8%) | 5 (4.0%) |  |
|  | Breast cancer | 14 (4.8%) | 2 (2.7%) | 7 (7.8%) | 5 (4.0%) |  |
|  | Prostate cancer | 12 (4.2%) | 0 (0%) | 6 (6.7%) | 6 (4.8%) |  |
|  | Cervical cancer (HSIL) | 12 (4.2%) | 0 (0%) | 3 (3.3%) | 9 (7.3%) |  |
|  | Colorectal cancer | 11 (3.8%) | 1 (1.3%) | 2 (2.2%) | 8 (6.5%) |  |
|  | Lung cancer | 9 (3.1%) | 2 (2.7%) | 3 (3.3%) | 4 (3.2%) |  |
|  | Malignant lymphoproliferative diseases | 8 (2.8%) | 0 (0%) | 4 (4.4%) | 4 (3.2%) |  |
|  | Bladder cancer | 7 (2.4%) | 0 (0%) | 3 (3.3%) | 4 (3.2%) |  |
|  | Melanoma | 6 (2.1%) | 0 (0%) | 0 (0%) | 6 (4.8%) |  |
|  | Other | 19 (6.6%) | 6 (8.0%) | 4 (4.4%) | 9 (7.3%) |  |
| **HIV diagnosis coincides with tumor diagnosis, n (%)** | Yes | 72 (24.9%) | 32 (42.7%) | 21 (23.3%) | 19 (15.3%) | **0.0001** |
|  | No | 217 (75.1%) | 43 (57.3%) | 69 (76.7%) | 105 (84.7%) |  |
| **Years HIV to cancer diagnosis** | Mean (SD) | 3.0 (6.4) | 0.5 (2.1) | 2.9 (5.2) | 6.7 (9.3) | **<0.0001** |
|  | Med [IQR] | 0.0 [0.0;3.2] | 0.0 [0.0;0.1] | 0.0 [0.0;4.3] | 1.3 [0.0;15.7] |  |
| **T CD4+ cell count at nadir, cell/mm^3^** | Mean (SD) | 205.7 (194.0) | 145.6 (147.9) | 204.2 (215.1) | 247.1 (195.5) | **0.0002** |
|  | Med [IQR] | 147.0 [70.0;290.0] | 90.0 [40.0;197.5] | 129.5 [60.0;270.0] | 200.0 [100.2;334.0] |  |
| **T CD4 + lymphocyte values (<200 CD4/mcL), n (%)** | Yes | 111 (40.7%) | 52 (70.3%) | 31 (38.8%) | 28 (23.5%) | **<0.0001** |
|  | No | 162 (59.3%) | 22 (29.7%) | 49 (61.3%) | 91 (76.5%) |  |
| **T CD4 + lymphocyte values (>500 CD4/mcL), n (%)** | Yes | 80 (29.3%) | 5 (6.8%) | 20 (25.0%) | 55 (46.2%) | **<0.0001** |
|  | No | 193 (70.7%) | 69 (93.2%) | 60 (75.0%) | 64 (53.8%) |  |
| **Undetectable viremia (< 50 cp/ml), n (%)** | Yes | 141 (48.8%) | 6 (8.0%) | 43 (47.8%) | 92 (74.2%) | **<0.0001** |
|  | No | 148 (51.2%) | 69 (92.0%) | 47 (52.2%) | 32 (25.8%) |  |
| **Risk factor, n (%)** | Blood product | 2 (0.7%) | 0 (0%) | 0 (0%) | 2 (1.6%) | 0.0908 |
|  | Unprotected heterosexual sex | 75 (26.0%) | 11 (14.7%) | 26 (28.9%) | 38 (30.6%) |  |
|  | Unprotected sex between men | 147 (50.9%) | 48 (64.0%) | 41 (45.6%) | 58 (46.8%) |  |
|  | Intravenous drug use | 64 (22.1%) | 16 (21.3%) | 22 (24.4%) | 26 (21.0%) |  |
|  | Vertical transmission | 1 (0.3%) | 0 (0%) | 1 (1.1%) | 0 (0%) |  |
| **In HAART at diagnosis, n (%)** | Yes | 188 (65.1%) | 28 (37.3%) | 61 (67.8%) | 99 (79.8%) | **<0.0001** |
|  | No | 101 (34.9%) | 47 (62.7%) | 29 (32.2%) | 25 (20.2%) |  |
| **Anticancer treatment, n (%)** | Yes | 273 (94.8%) | 73 (97.3%) | 82 (91.1%) | 118 (95.9%) | 0.1905 |
|  | No | 15 (5.2%) | 2 (2.7%) | 8 (8.9%) | 5 (4.1%) |  |
| **Chemotherapy, n (%)** | Yes | 135 (47.9%) | 39 (52.0%) | 43 (47.8%) | 53 (45.3%) | 0.6627 |
|  | No | 147 (52.1%) | 36 (48.0%) | 47 (52.2%) | 64 (54.7%) |  |
| **Radiotherapy, n (%)** | Yes | 50 (17.7%) | 13 (17.3%) | 20 (22.2%) | 17 (14.5%) | 0.3544 |
|  | No | 232 (82.3%) | 62 (82.7%) | 70 (77.8%) | 100 (85.5%) |  |
| **Surgical treatment, n (%)** | Yes | 115 (40.5%) | 11 (14.7%) | 36 (40.0%) | 68 (57.1%) | **<0.0001** |
|  | No | 169 (59.5%) | 64 (85.3%) | 54 (60.0%) | 51 (42.9%) |  |
| **Tumor outcome, n (%)** | Progression | 71 (24.9%) | 19 (25.3%) | 23 (25.8%) | 29 (24.0%) | 0.0515 |
|  | Complete remission | 171 (60.0%) | 41 (54.7%) | 61 (68.5%) | 69 (57.0%) |  |
|  | Partial remission | 43 (15.1%) | 15 (20.0%) | 5 (5.6%) | 23 (19.0%) |  |
| **2-year survival, n (%)** | Yes | 233 (80.9%) | 50 (66.7%) | 74 (82.2%) | 109 (88.6%) | **0.0006** |
|  | No | 55 (19.1%) | 25 (33.3%) | 16 (17.8%) | 14 (11.4%) |  |
| **10-year survival, n (%)** | Yes | 210 (72.9%) | 45 (60.0%) | 61 (67.8%) | 104 (84.6%) | **0.0003** |
|  | No | 78 (27.1%) | 30 (40.0%) | 29 (32.2%) | 19 (15.4%) |  |
| **Follow-up time** | Mean (SD) | 6.0 (6.0) | 9.4 (8.3) | 7.4 (5.0) | 3.0 (2.7) | **<0.0001** |
|  | Med [IQR] | 3.3 [1.2;9.7] | 9.6 [0.5;17.6] | 7.4 [3.3;11.0] | 2.3 [1.5;3.6] |  |
| **Follow-up person-years** | Total | 1731.7 | 704.3 | 662.3 | 365.1 |  |

**Table S2.** Characteristics of the study population, cancer treatment and outcome by tumor type and decade of cancer diagnosis (all proportions are intended as column percentage).

| **Characteristics** | | **ADC N = 120** | | | | **NADC N = 166** | | | | **p-value differences ADC VS NADC by decade** | | |
| --- | --- | --- | --- | --- | --- | --- | --- | --- | --- | --- | --- | --- |
|  |  | **First decade (1996 - 2003) N = 58** | **Second decade (2004 - 2013) N = 35** | **Third decade (2014 - 2023) N = 27** | **p-value** | **First decade (1996 - 2003) N = 17** | **Second decade (2004 - 2013) N = 55** | **Third decade (2014 - 2023) N = 94** | **p-value** | **First decade (1996 - 2003)** | **Second decade (2004 - 2013)** | **Third decade (2014 - 2023)** |
| **Age (at cancer diagnosis)** | mean (SD) | 41.4 (10.4) | 48.0 (10.0) | 44.8 (10.5) | **0.0040** | 42.4 (10.5) | 50.2 (11.1) | 57.1 (10.7) | **<0.0001** | 0.7371 | 0.8165 | **<0.0001** |
|  | Med [IQR] | 39.0 [33.2;45.0] | 49.0 [42.0;54.0] | 44.0 [40.0;46.0] |  | 40.0 [34.0;45.0] | 48.0 [42.5;56.0] | 57.0 [50.2;64.0] |  |  |  |  |
| **Sex, n (%)** | Female | 8 (13.8%) | 3 (8.6%) | 2 (7.4%) | 0.6947 | 2 (11.8%) | 19 (34.5%) | 29 (30.9%) | 0.1962 | 1.0000 | **0.0052** | **0.0139** |
|  | Male | 50 (86.2%) | 32 (91.4%) | 25 (92.6%) |  | 15 (88.2%) | 36 (65.5%) | 65 (69.1%) |  |  |  |  |
| **Nationality, n (%)** | Italian | 52 (89.7%) | 33 (94.3%) | 22 (81.5%) | 0.2783 | 17 (100.0%) | 51 (92.7%) | 86 (91.5%) | 0.7417 | 0.3266 | 1.0000 | 0.1619 |
|  | Foreign | 6 (10.3%) | 2 (5.7%) | 5 (18.5%) |  | 0 (0%) | 4 (7.3%) | 8 (8.5%) |  |  |  |  |
| **Tumor, n (%)** | Kaposi | 30 (51.7%) | 18 (51.4%) | 15 (55.6%) | 0.9365 | 0 (0%) | 0 (0%) | 0 (0%) | **0.0009** | **<0.0001** | **<0.0001** | **<0.0001** |
|  | NHL | 28 (48.3%) | 17 (48.6%) | 12 (44.4%) |  | 0 (0%) | 1 (1.8%) | 0 (0%) |  |  |  |  |
|  | HCC | 0 (0%) | 0 (0%) | 0 (0%) |  | 1 (5.9%) | 7 (12.7%) | 13 (13.8%) |  |  |  |  |
|  | Anal cancer | 0 (0%) | 0 (0%) | 0 (0%) |  | 2 (11.8%) | 5 (9.1%) | 11 (11.7%) |  |  |  |  |
|  | Non-melanoma skin cancer | 0 (0%) | 0 (0%) | 0 (0%) |  | 1 (5.9%) | 3 (5.5%) | 12 (12.8%) |  |  |  |  |
|  | Neoplasms of the oral cavity, pharynx, and salivary glands | 0 (0%) | 0 (0%) | 0 (0%) |  | 2 (11.8%) | 7 (12.7%) | 5 (5.3%) |  |  |  |  |
|  | Breast cancer | 0 (0%) | 0 (0%) | 0 (0%) |  | 2 (11.8%) | 7 (12.7%) | 5 (5.3%) |  |  |  |  |
|  | Prostate cancer | 0 (0%) | 0 (0%) | 0 (0%) |  | 0 (0%) | 6 (10.9%) | 6 (6.4%) |  |  |  |  |
|  | Cervical cancer (HSIL) | 0 (0%) | 0 (0%) | 0 (0%) |  | 0 (0%) | 3 (5.5%) | 8 (8.5%) |  |  |  |  |
|  | Colorectal cancer | 0 (0%) | 0 (0%) | 0 (0%) |  | 1 (5.9%) | 2 (3.6%) | 8 (8.5%) |  |  |  |  |
|  | Lung cancer | 0 (0%) | 0 (0%) | 0 (0%) |  | 2 (11.8%) | 3 (5.5%) | 3 (3.2%) |  |  |  |  |
|  | Malignant lymphoproliferative diseases | 0 (0%) | 0 (0%) | 0 (0%) |  | 0 (0%) | 4 (7.3%) | 4 (4.3%) |  |  |  |  |
|  | Bladder cancer | 0 (0%) | 0 (0%) | 0 (0%) |  | 0 (0%) | 3 (5.5%) | 4 (4.3%) |  |  |  |  |
|  | Melanoma | 0 (0%) | 0 (0%) | 0 (0%) |  | 0 (0%) | 0 (0%) | 6 (6.4%) |  |  |  |  |
|  | Other | 0 (0%) | 0 (0%) | 0 (0%) |  | 6 (35.3%) | 4 (7.3%) | 9 (9.6%) |  |  |  |  |
| **HIV diagnosis coincides with tumor diagnosis, n (%)** | Yes | 31 (53.4%) | 21 (60.0%) | 19 (70.4%) | 0.3332 | 1 (5.9%) | 0 (0%) | 0 (0%) | 0.1024 | **0.0005** | **<0.0001** | **<0.0001** |
|  | No | 27 (46.6%) | 14 (40.0%) | 8 (29.6%) |  | 16 (94.1%) | 55 (100.0%) | 94 (100.0%) |  |  |  |  |
| **Years HIV to cancer diagnosis** | Mean (SD) | 0.4 (1.2) | 0.7 (3.0) | 0.6 (3.6) | 0.7820 | 1.2 (5.2) | 7.7 (5.8) | 13.4 (9.1) | **0.0010** | 0.1960 | **<0.0001** | **<0.0001** |
|  | Med [IQR] | 0.0 [0.0;0.0] | 0.0 [0.0;0.0] | 0.0 [0.0;0.0] |  | 1.8 [-0.8;4.0] | 4.4 [3.3;12.3] | 15.5 [4.0;20.5] |  |  |  |  |
| **T CD4+ cell count at nadir, cell/mm^3^** | Mean (SD) | 141.7 (156.8) | 168.7 (160.1) | 139.3 (114.5) | 0.5522 | 162.9 (102.5) | 229.2 (245.1) | 282.1 (205.4) | **0.0150** | 0.1218 | 0.4142 | **0.0004** |
|  | Med [IQR] | 84.5 [30.0;198.8] | 100.0 [40.0;260.0] | 115.0 [60.0;193.0] |  | 135.0 [90.0;190.0] | 140.0 [60.0;280.0] | 246.5 [130.2;387.5] |  |  |  |  |
| **T CD4 + lymphocyte values (<200 CD4/mcL), n (%)** | Yes | 41 (70.7%) | 20 (62.5%) | 16 (64.0%) | 0.6868 | 11 (68.8%) | 11 (22.9%) | 11 (12.1%) | **<0.0001** | 1.0000 | **0.0004** | **<0.0001** |
|  | No | 17 (29.3%) | 12 (37.5%) | 9 (36.0%) |  | 5 (31.2%) | 37 (77.1%) | 80 (87.9%) |  |  |  |  |
| **T CD4 + lymphocyte values (>500 CD4/mcL), n (%)** | Yes | 3 (5.2%) | 4 (12.5%) | 3 (12.0%) | 0.3443 | 2 (12.5%) | 16 (33.3%) | 49 (53.8%) | **0.0022** | 0.2941 | **0.0350** | **0.0002** |
|  | No | 55 (94.8%) | 28 (87.5%) | 22 (88.0%) |  | 14 (87.5%) | 32 (66.7%) | 42 (46.2%) |  |  |  |  |
| **Undetectable viremia (< 50 cp/ml), n (%)** | Yes | 1 (1.7%) | 10 (28.6%) | 8 (29.6%) | **<0.0001** | 5 (29.4%) | 33 (60.0%) | 82 (87.2%) | **<0.0001** | **0.0018** | **0.0036** | **<0.0001** |
|  | No | 57 (98.3%) | 25 (71.4%) | 19 (70.4%) |  | 12 (70.6%) | 22 (40.0%) | 12 (12.8%) |  |  |  |  |
| **Risk factor, n (%)** | Blood product | 0 (0%) | 0 (0%) | 0 (0%) | 0.3345 | 0 (0%) | 0 (0%) | 2 (2.1%) | 0.6198 | 0.2021 | **0.0449** | **0.0025** |
|  | Unprotected heterosexual sex | 8 (13.8%) | 10 (28.6%) | 4 (14.8%) |  | 3 (17.6%) | 16 (29.1%) | 33 (35.1%) |  |  |  |  |
|  | Unprotected sex between men | 40 (69.0%) | 21 (60.0%) | 21 (77.8%) |  | 8 (47.1%) | 20 (36.4%) | 35 (37.2%) |  |  |  |  |
|  | Intravenous drug use | 10 (17.2%) | 4 (11.4%) | 2 (7.4%) |  | 6 (35.3%) | 18 (32.7%) | 24 (25.5%) |  |  |  |  |
|  | Vertical transmission | 0 (0%) | 0 (0%) | 0 (0%) |  | 0 (0%) | 1 (1.8%) | 0 (0%) |  |  |  |  |
| **In HAART at diagnosis, n (%)** | Yes | 16 (27.6%) | 18 (51.4%) | 8 (29.6%) | 0.0524 | 12 (70.6%) | 43 (78.2%) | 88 (93.6%) | **0.0030** | **0.0013** | **0.0081** | **<0.0001** |
|  | No | 42 (72.4%) | 17 (48.6%) | 19 (70.4%) |  | 5 (29.4%) | 12 (21.8%) | 6 (6.4%) |  |  |  |  |
| **Anticancer treatment, n (%)** | Yes | 56 (96.6%) | 34 (97.1%) | 24 (92.3%) | 0.7062 | 17 (100.0%) | 48 (87.3%) | 91 (96.8%) | 0.0507 | 1.0000 | 0.1435 | 0.2959 |
|  | No | 2 (3.4%) | 1 (2.9%) | 2 (7.7%) |  | 0 (0%) | 7 (12.7%) | 3 (3.2%) |  |  |  |  |
| **Chemotherapy, n (%)** | Yes | 27 (46.6%) | 22 (62.9%) | 20 (76.9%) | **0.0262** | 12 (70.6%) | 21 (38.2%) | 32 (36.4%) | **0.0284** | 0.0811 | **0.0223** | **0.0003** |
|  | No | 31 (53.4%) | 13 (37.1%) | 6 (23.1%) |  | 5 (29.4%) | 34 (61.8%) | 56 (63.6%) |  |  |  |  |
| **Radiotherapy, n (%)** | Yes | 8 (13.8%) | 9 (25.7%) | 1 (3.8%) | 0.0618 | 5 (29.4%) | 11 (20.0%) | 16 (18.2%) | 0.5233 | 0.1552 | 0.5250 | 0.1140 |
|  | No | 50 (86.2%) | 26 (74.3%) | 25 (96.2%) |  | 12 (70.6%) | 44 (80.0%) | 72 (81.8%) |  |  |  |  |
| **Surgical treatment, n (%)** | Yes | 3 (5.2%) | 2 (5.7%) | 0 (0%) | 0.6033 | 8 (47.1%) | 34 (61.8%) | 66 (73.3%) | 0.0698 | **0.0002** | **<0.0001** | **<0.0001** |
|  | No | 55 (94.8%) | 33 (94.3%) | 26 (100.0%) |  | 9 (52.9%) | 21 (38.2%) | 24 (26.7%) |  |  |  |  |
| **Tumor outcome, n (%)** | Progression | 13 (22.4%) | 5 (14.3%) | 5 (19.2%) | **0.0444** | 6 (35.3%) | 18 (33.3%) | 24 (26.1%) | 0.1264 | 0.1650 | 0.0571 | 0.4466 |
|  | Complete remission | 35 (60.3%) | 29 (82.9%) | 14 (53.8%) |  | 6 (35.3%) | 32 (59.3%) | 53 (57.6%) |  |  |  |  |
|  | Partial remission | 10 (17.2%) | 1 (2.9%) | 7 (26.9%) |  | 5 (29.4%) | 4 (7.4%) | 15 (16.3%) |  |  |  |  |
| **2-year survival, n (%)** | Yes | 39 (67.2%) | 29 (82.9%) | 22 (81.5%) | 0.1637 | 11 (64.7%) | 45 (81.8%) | 84 (90.3%) | **0.0205** | 0.8454 | 0.9000 | **0.3034** |
|  | No | 19 (32.8%) | 6 (17.1%) | 5 (18.5%) |  | 6 (35.3%) | 10 (18.2%) | 9 (9.7%) |  |  |  |  |
| **10-year survival, n (%)** | Yes | 38 (65.5%) | 25 (71.4%) | 22 (81.5%) | 0.3196 | 7 (41.2%) | 36 (65.5%) | 79 (84.9%) | **0.0002** | 0.0716 | 0.5544 | **0.7650** |
|  | No | 20 (34.5%) | 10 (28.6%) | 5 (18.5%) |  | 10 (58.8%) | 19 (34.5%) | 14 (15.1%) |  |  |  |  |
| **Follow-up time** | Mean (SD) | 9.9 (8.3) | 7.2 (4.6) | 2.8 (3.9) | 0.0035 | 7.7 (8.5) | 7.5 (5.3) | 3.1 (2.3) | **<0.0001** | 0.6622 | 0.8783 | **0.1369** |
|  | Med [IQR] | 13.3 [0.5;17.6] | 7.7 [4.7;10.2] | 2.2 [0.5;3.0] |  | 2.7 [0.4;16.5] | 7.2 [2.7;11.2] | 2.5 [1.7;3.9] |  |  |  |  |
